# ‘Maisons Des Adolescents’, Youth Mental Health in France

**DOI:** 10.1192/j.eurpsy.2022.159

**Published:** 2022-09-01

**Authors:** L. Benoit

**Affiliations:** Yale University, Child Study Center, New Haven, United States of America

**Keywords:** integrated youth health care services, Maison des Adolescents, mental health, transition

## Abstract

Since 1999, more than 100 “Maisons des Adolescents” (MDAs) - “House of Adolescents” - have been developed in France. These integrated youth-friendly facilities enabled young persons to gain access to specific care. The various medical programs of MDAs depend on the priorities of local communities rather than on official regulations. Most MDAs offer the following essential services: a “Health and Prevention Space” open daily; multidisciplinary consultations; consult liaison for youths hospitalized in medical units; a home visiting service; outpatient clinic including art workshops; refresher courses for school work; peer and parent support groups. The MDAs from the start addressed an age group (young people aged 11-21 years) rather than an illness. They thus provide primary prevention for young persons according to the World Health Organization definition of health as “a state of complete physical, mental and social well-being.” The success of the MDA network is already widely acknowledged by users, professionals, and policymakers.

**Disclosure:**

No significant relationships.

